# One Disorder, Two Brains: Challenges in Modeling Epilepsy and Associated Movement Disorders in Mice

**DOI:** 10.3390/ijms27219916

**Published:** 2026-11-05

**Authors:** Daniil I. Travin, Polina E. Anisimova, Ksenia A. Bogovarova, Angelina O. Kustova, Victor S. Tarabykin

**Affiliations:** 1 Institute of Neuroscience, Lobachevsky State University of Nizhny Novgorod, 603022 Nizhny Novgorod, Russia; 2 Department of Genetics and Life Sciences, Sirius University of Science and Technology, 354340 Sochi, Russia

**Keywords:** phenotypic recapitulation, molecular redundancy, ortholog heterotopia, translational barriers

## Abstract

Modeling epileptic syndromes co-occurring with movement disorders in mice often fails to fully recapitulate human phenotypes due to distinct gene-specific and anatomical mechanisms. This review categorizes these translational barriers into three primary groups. First, ortholog heterotopia and heterochrony drive spatiotemporal shifts in gene expression, causing orthologs to activate at disparate developmental stages or within different cell populations across species. Second, molecular redundancy and compensation obscure phenotypes through the upregulation of alternative proteins in response to target protein suppression, the activation of modifier genes, or neural network rewiring. Third, structural variations in striatal anatomy fundamentally alter corticostriatal loop functioning. Characterizing these divergent mechanisms is essential for improving the predictive validity of preclinical models and advancing targeted therapies.

## 1. Introduction

Many genes associated with epilepsy are involved in regulating neuronal excitability, synaptic transmission, and the functioning of ion channels. Their disruption can affect several systems at once and lead not only to epilepsy but also to motor disorders [1,2]. For example, disruption of *SCN1A* affects the excitability of GABAergic interneurons, which contributes to the development of epilepsy, but the clinical picture is not limited to seizures: motor disorders may also be observed in Dravet syndrome [3,4,5]. Therefore, if we evaluate the model solely based on the presence of seizures, we may fail to notice other important consequences of the same mutation.

Furthermore, the manifestation of one phenotype can make it difficult to interpret another [6]. Suppose we have obtained a model in which seizures and motor disorders are observed. Several questions arise: is the motor disorder a consequence of the same mutation [6], or could it be the result of recurring seizures [7], or perhaps the motor deviation appeared as a result of treatment with antiepileptic drugs, if such treatment was carried out [8]?

To investigate the mechanisms underlying the pathogenesis of such disorders, novel transgenic lines modeling patient-identified mutations have been generated [9]. Mouse models of human diseases are widely utilized in laboratory practice [10] due to interspecies physiological conservation and cost-effectiveness [9]. However, these models often fail to fully recapitulate the entire spectrum of human clinical features. Often, animals reproduce only part of the clinical picture: for instance, a model may exhibit typical seizures but lack the characteristic motor disorders, or vice versa [11,12]. Such discrepancies help us understand the limitations of a particular model—specifically, the gap between genetic construct validity and phenotypic face validity—and highlight important species-specific differences. This discordance stems from a range of genetic, molecular, and anatomical factors that modulate symptom severity [13].

**Figure 1:**
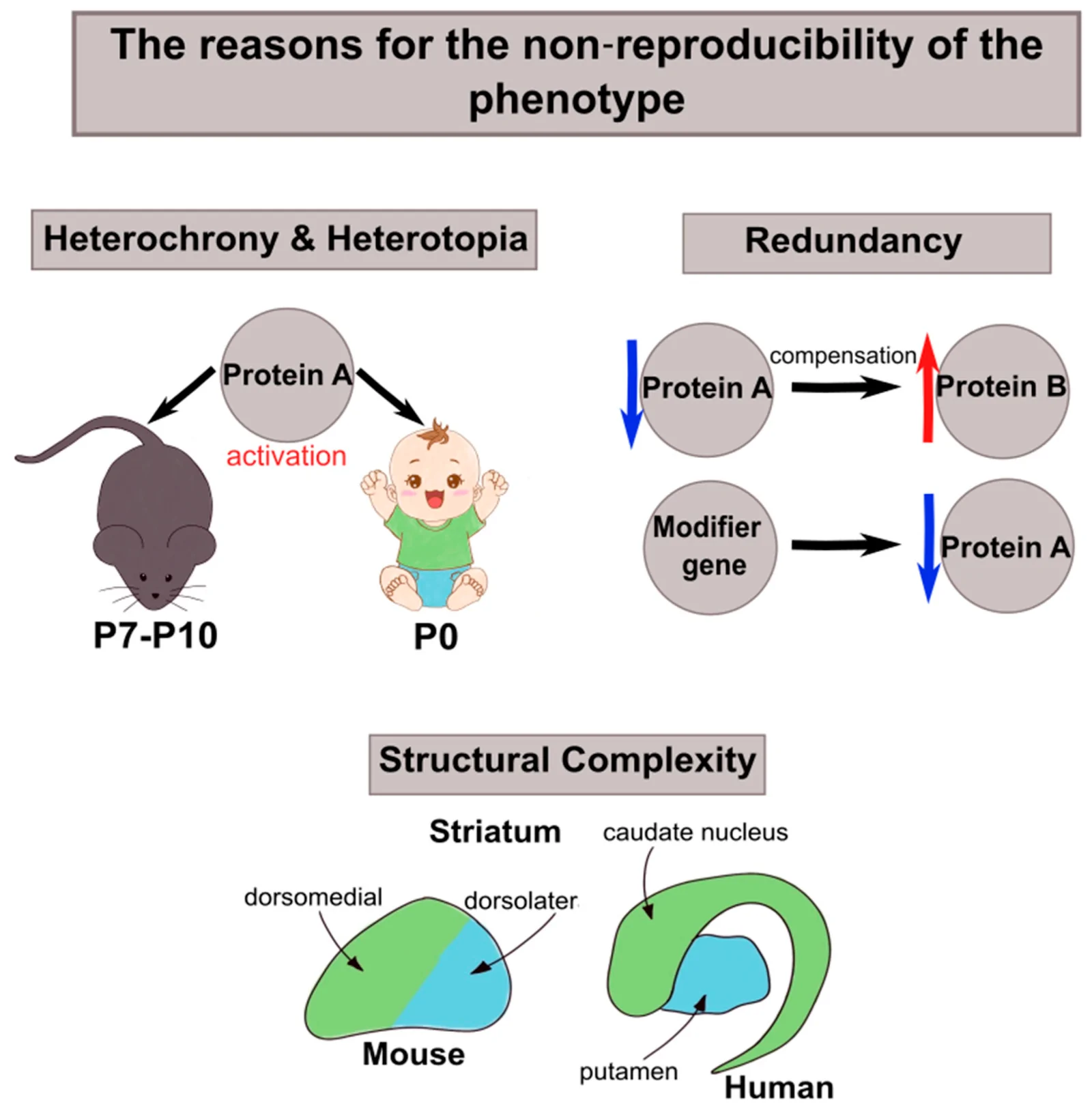
Mechanisms underlying phenotypic deviations in mouse models. (Red arrows indicate expression upregulation; blue arrows indicate expression downregulation.) Three main mechanisms contribute to the failure of mouse models to fully recapitulate the phenotype of epilepsy co-occurring with movement disorders. First, spatiotemporal shifts in gene expression exist between mice and humans, meaning that specific genes are activated at distinct developmental stages and neuroanatomical locations across species. Second, molecular redundancy and functional compensation can occur, leading either to the compensatory upregulation of alternative proteins in response to target protein suppression, or to the activation of modifier genes that modulate mutant protein expression. Third, structural variations in striatal anatomy lead to functional differences in corticostriatal loops between humans and rodents.

In this review, we describe experimentally validated, gene-specific mechanisms that complicate the modeling of epileptic syndromes co-occurring with movement disorders in mouse models. We have categorized these known mechanisms into three main groups: ortholog heterotopia and heterochrony, molecular redundancy and compensation, and structural variations in striatal anatomy (Figure 1). The first group is driven by temporal and spatial shifts in ortholog expression between mice and humans. This implies that the activation of a specific gene in these species occurs at distinct developmental stages or within different cell populations [14]. The second group is associated with mechanisms of molecular redundancy and compensation, manifested as the upregulation of other proteins in response to the suppression of a given protein, activation of modifier genes, and neural network rewiring [15,16,17]. The third mechanism particularly impacts the motor features of the phenotype and is associated with structural variations in striatal anatomy and, consequently, in the functioning of corticostriatal loops between mice and humans. The human dorsal striatum is anatomically divided into the caudate nucleus and putamen, whereas in the mouse it forms a single contiguous structure [18]. Furthermore, humans possess robust connectivity between the dorsolateral prefrontal cortex and the striatum, which is absent in rodents. Despite substantial similarities in limbic and sensorimotor corticostriatal networks, the associative circuits subserving human cognitive behavior differ markedly from that of the rodents [19].

Investigating the factors that influence the phenotype of animal models allows not only for a better understanding of the translational limitations of transgenic mouse models, but also for identifying novel potential mechanisms governing brain adaptation to pathogenic gene variants. This is essential for proper experimental design and the accurate interpretation of data obtained from transgenic models.

## 2. Temporal and Spatial Shifts in Gene Expression

A high degree of ortholog conservation does not inherently guarantee phenotypic congruence between humans and mice. A single gene may be developmentally activated across distinct brain regions or within disparate postnatal developmental windows in these two species. Consequently, a mutation critical to a specific neuronal population in humans may operate within a markedly different molecular and neuroanatomical context in rodents [14].

### 2.1. Heterotopia: Spatial Shifts in Gene Expression

The GluN2D subunit, encoded by the *GRIN2D* gene, is a component of N-methyl-D-aspartate receptors (NMDARs)—ionotropic glutamate receptors expressed predominantly in GABAergic interneurons of the cortex, hippocampus, and basal ganglia. GluN2D-containing NMDARs are characterized by low sensitivity to Mg^2+^ voltage-dependent block and slow deactivation kinetics, enabling their activation at depolarization levels where other NMDAR subtypes remain blocked [20,21]. In humans, gain-of-function (GoF) variants in *GRIN2D* cause severe developmental and epileptic encephalopathy (DEE). The clinical spectrum features refractory seizures, severe axial hypotonia, neurodevelopmental delay, and motor automatisms [22]. Heterozygous knock-in mutant mice recapitulate certain behavioral, neuroanatomical, and electrophysiological abnormalities: lethal convulsive seizures initiate at postnatal day (P) 17, and prolonged bursts of spike-wave discharges (SWDs) are recorded in adult animals [22].

However, GluN2D expression dynamics reveal potential limitations of this model. In mice, GluN2D expression is prominent in the neonatal brainstem and diencephalon. In adult mice, it is restricted to specific cerebellar nuclei, the striatum, olfactory bulb, nigral dopaminergic neurons, subthalamic nucleus, and hippocampal interneurons [23]. In adult mice, the striatal GluN2D pool is substantially diminished relative to the prenatal period, whereas humans maintain significant expression within the striatum and interconnected motor structures throughout adulthood. This regional population represents the likely anatomical substrate for motor impairment in *GRIN2D*-DEE [20,24,25].

Because GluN2D expression in the adult mouse striatum is minimal, structural defects (enlarged presynaptic terminals and increased synaptic cleft width) develop predominantly in the cerebellum and hippocampus rather than in striatal motor circuits. As a result, specific motor automatisms or dyskinesias—which form the core clinical phenotype of *GRIN2D*-DEE in humans—failed to be recapitulated in mice [22]. Notably, pharmacological interventions demonstrated limited efficacy in normalizing pathological brain activity in *Grin2d* mutant mice, and ketamine, which exerts anticonvulsant effects in patients with *GRIN2D*-DEE, exhibited proconvulsant properties in mutant [26]. It should be noted, however, that this pharmacological discrepancy may partly reflect differences in dosing, route of administration, and experimental context rather than a straightforward species-specific effect.

### 2.2. Heterochrony: Temporal Shifts in Gene Expression

*GRIN2A* encodes the GluN2A subunit of the ionotropic NMDAR. NMDARs are excitatory ionotropic glutamate receptors mediating Na^+^ and Ca^2+^ influx and K^+^ efflux, playing fundamental roles in synaptic transmission, plasticity, brain development, learning, and memory [20,27,28]. A heterozygous GoF variant in *GRIN2A* was reported in a human patient with severe DEE and myoclonic seizures, infantile spasms, and subsequent pharmacoresistant tonic–clonic seizures [29]. Mouse models mirror the molecular GoF effect and associated alterations in neuronal excitability but fail to fully reproduce the primary clinical phenotype: in humans, heterozygous variants are associated with severe spontaneous epilepsy, whereas knock-in mouse models display spontaneous seizures only in homozygous animals [29,30]. Furthermore, alterations in induced seizure thresholds in heterozygous mice were bidirectional depending on the paradigm, arguing against a simple uniform increase in seizure susceptibility [29]. The behavioral phenotype of these mice also only partially recapitulated human disease manifestations, exhibiting hyperactivity, stereotypies, and mild gait or motor coordination deficits [29].

It appears that a single mutant allele in mice is sufficient to alter local network activity but insufficient to trigger a transition to spontaneous epilepsy. This discrepancy is likely modulated by cell-type-specific circuit architecture [31] and genetic background [29,32], as well as age and sex [33]. Additionally, the magnitude of the GluN2A(S644G) GoF effect depends on receptor subunit composition. In the heterozygous state, mutant GluN2A can co-assemble either with wild-type GluN2A or with GluN2B [34]; in tri-heteromeric assemblies containing GluN2B, the functional impact of the mutation is blunted [29,34]. Crucially, the postnatal expression ratio of GluN2A to GluN2B progressively increases [20,35]. In mice, this developmental “GluN2B-to-GluN2A switch” occurs rapidly over 2–5 weeks [36], whereas in humans, it spans several years [37]. Consequently, in mice, the pathological effect of the mutation manifests rapidly during a stage when neural networks retain high developmental plasticity, enabling potential functional compensation. In contrast, the maturing human neural network progressively accumulates mutation-sensitive GluN2A-containing receptors over an extended period, potentially creating a permissive environment for progressive hyperexcitability and the emergence of spontaneous epilepsy.

*SCN8A* encodes the voltage-gated sodium channel Nav1.6, the principal ion channel at the axon initial segment and nodes of Ranvier in mammals [38]. The spectrum of *SCN8A*-related disorders encompasses DEE with severe developmental delay, juvenile pharmacoresistant epilepsy, and motor disturbances including dystonia, ataxia, and choreoathetosis [39,40]. Early knock-in lines harbouring patient-derived GoF variants reproduced seizures and premature lethality but failed to recapitulate motor abnormalities [38,39]. Recapitulation of the full symptom spectrum required the generation of a line with premature Nav1.6 activation—the only mechanism that enabled simultaneous reproduction of both epileptic and motor manifestations [38]. When expression was restricted to excitatory forebrain neurons, the motor phenotype was abolished, indicating divergent neuronal substrates underlying the disease in humans and mice. The mRNA splicing pattern of *SCN2A*, *SCN3A*, and *SCN8A* undergoes developmental regulation in the brain: a neonatal copy of the fifth coding exon (5N) is included perinatally, whereas an alternative mutually exclusive copy (5A) predominates in the mature brain [41]. For all three genes, the coordinated transition from 5N to 5A occurs between gestational week 24 and six years of age in humans, whereas the equivalent transition in mice begins no later than embryonic day E15.5. This temporal shift results in mutations acting on distinct channel isoforms at non-overlapping stages of neural circuit maturation. In addition to the 5N/5A pair, *SCN8A* harbours a second set of mutually exclusive exons—18N and 18A [42]. During early fetal development, transcripts containing 18N or lacking both exons predominate; in humans, the transition to 18A-containing transcripts occurs between gestational week 13 and six months of postnatal life [41]. In mice, the proportion of 18N-containing transcripts peaks between E12.5 and P1.5, after which 18A rapidly becomes dominant [41,43]. The earlier and more compressed transition in mice substantially narrows the window of fetal isoform expression. Consequently, standard GoF mutations in the murine ontogenetic context do not engage the same stages of motor circuit maturation that are affected in humans. Only a mutation that shifts the timing of channel activation compensates for this ontogenetic divergence and reproduces the full phenotypic spectrum [38,41,43].

However, it should be noted that the human–mouse difference in the timing of the 18N-to-18A switch is not yet firmly established as the primary driver of motor phenotype discrepancies. Some standard GoF knock-in models do display mild motor abnormalities, and the mutations used across studies (N1768D, R1872W versus the premature-activation T767I allele) have distinct biophysical effects that provide an equally plausible explanation for the observed differences in phenotypic completeness. Genetic background and the extent of motor phenotyping may also contribute [38,41,43]. Thus, while splicing heterochrony remains a reasonable candidate modifier, further direct comparative evidence is required.

Heterochrony does not always lead to a milder phenotype in mice. It can even exacerbate it, as in the case of *SNAP25*. The *SNAP25* gene encodes the SNAP-25 protein, a core component of the SNARE complex that mediates Ca2+-dependent synaptic vesicle fusion with the presynaptic membrane and subsequent neurotransmitter release [44,45]. A patient with the *SNAP25* L50S variant (a missense mutation resulting in the substitution of a hydrophobic leucine for a polar serine at position 50 of the protein) presented with infantile spasms, tonic seizures with an onset at 7 months of age, and developmental delay [46,47]. However, heterozygous mice carrying the exact same amino acid substitution (*Snap25* L50S) exhibit a much more severe phenotype. In addition to recurrent focal and generalized tonic–clonic seizures, these mice develop profound motor and behavioral deficits, impaired long-term memory, and experience high early postnatal mortality [48]. Such striking phenotypic differences between humans and mice may be attributed to several factors. First, species-specific timelines of brain maturation directly influence the capacity of neural networks to adapt to persistent disruptions in synaptic transmission [49,50]. The protracted maturation of the human brain provides a broader window for engaging homeostatic mechanisms, allowing for the fine-tuning of synaptic strength and intrinsic neuronal excitability [51]. Ultimately, the rarity of the L50S variant limits broader clinical conclusions [46]. It should be noted that, along with the L50S mutation, the authors also introduced two synonymous substitutions (G43 and R45). Although they do not change the amino acid sequence, they could potentially lead to undesirable disruptions at the RNA level [48].

### 2.3. Combined Spatiotemporal Divergence

The *UBE3A* gene encodes ubiquitin-protein ligase E3A (E6-AP), a HECT domain-containing E3 ligase within the ubiquitin-proteasome system. *UBE3A* mediates targeted protein degradation via the proteasomal pathway through ubiquitin labeling [52]. At the molecular level, it regulates synaptic plasticity, transcriptional control, and circadian rhythmicity [53]. A hallmark feature of *UBE3A* is its neuron-specific genomic imprinting: in neurons, the paternal allele is predominantly silenced through an epigenetic mechanism. Consequently, *UBE3A* exhibits maternal allele-specific expression across most brain regions in both mice and humans, with the highest expression observed in hippocampal and cerebellar neurons [54,55].

Mutations in *UBE3A* are causal for Angelman syndrome (AS), a rare neurodevelopmental disorder [54]. Patients with AS present with severe intellectual disability, absence of speech, epilepsy, characteristic dysmorphic facial features, ataxia, and a distinct behavioral phenotype including hyperactivity, short attention span, and frequent sleep disruptions [54]. Maternal-deficient (*Ube3a*^m−/p+^) mice recapitulate microcephaly, ataxia, motor deficits, EEG abnormalities, and audiogenic seizure susceptibility.

However, imprinting is incomplete and regionally heterogeneous in both species. In the mouse *Ube3a*^m−/p+^ Angelman model, paternal UBE3A protein is not completely silenced: it persists in a spatially and cell-type-restricted mosaic pattern, most notably in neurons of the suprachiasmatic nucleus (SCN) that co-express arginine vasopressin and core clock proteins PER2 and BMAL1 [56]. In humans, this process is significantly prolonged: in cerebral organoids, *UBE3A* still exhibits robust biallelic (including nuclear) expression at 3–7 weeks of differentiation, with progressive paternal allele silencing becoming evident after approximately 6 weeks and continuing through weeks 10–17 [57,58]. Thus, in humans the paternal allele remains functionally active across a substantially longer window of neuronal differentiation. The *Ube3a*^m−/p+^ mouse model therefore does not fully capture the extended developmental window of biallelic expression or the precise human spatiotemporal pattern of residual paternal protein, thereby contributing to incomplete phenotypic recapitulation.

*DEPDC5* encodes a component of the GATOR1 complex, which—together with NPRL2 and NPRL3—functions as a negative regulator of mTORC1, suppressing its activity under amino acid-deficient conditions [59]. In humans, heterozygous pathogenic variants in *DEPDC5* are associated with a broad phenotypic spectrum that includes structural cortical abnormalities (focal cortical dysplasia and hemimegalencephaly), focal epilepsy, and marked incomplete penetrance: a substantial fraction of carriers remain asymptomatic. Mosaic second-hit mutations have been identified in some patients, including occasionally in individuals without clinical manifestations [60,61,62,63]. However, heterozygous *Depdc5*^+^/^−^ mice—carrying the same genotype as affected patients—typically do not develop spontaneous seizures or overt cortical dysplasia [64,65,66,67]. This discrepancy is most likely multifactorial. A “two-hit” model has been proposed for *DEPDC5*-related epilepsy [61,62], in which the inherited heterozygous mutation constitutes the first “hit”—establishing disease susceptibility but being insufficient on its own to generate an epileptogenic cortical lesion [61]. The second “hit” is caused by a stochastic somatic mutation arising in a small population of progenitor cells during brain development or, possibly, postnatally [63]. Should this mutation inactivate the remaining wild-type *DEPDC5* allele, a focal clone of cells with biallelic loss of function emerges; it is this mosaic clone—rather than systemic heterozygosity per se—that is thought to drive pathological mTORC1 activation and epileptogenic focus formation [61,68]. The standard heterozygous *Depdc5*^+^/^−^ mouse recapitulates only the first hit [61,63]. The probability of a second “hit” is determined by the scale of cortical neurogenesis. In humans, cortical neurogenesis spans approximately four months—compared with approximately one week to eleven days in mice—generating on the order of 16 billion neocortical neurons and sustaining large proliferative progenitor pools, including outer radial glia (oRG), which are only sparsely represented in the murine brain [69,70,71,72,73]. The substantially fewer cell divisions and the abbreviated existence of proliferating progenitor populations in mice markedly reduce the probability that a somatic second-hit mutation will arise and give rise to a sufficiently large mosaic clone to initiate pathological processes [74,75,76,77].

In addition, the well-documented incomplete penetrance and variable expressivity of heterozygous *DEPDC5* variants in humans further complicate direct phenotypic comparisons. Thus, both the two-hit requirement (with its inherent stochasticity and incomplete penetrance) and species differences in neurogenic scale contribute to the limited recapitulation observed in heterozygous mouse models.

## 3. Molecular Redundancy and Compensation

Genetic and functional compensation can be mediated by paralogs or other functionally related proteins [16,78], alterations in ionic currents [15], and developmental adaptations within neural networks [17]. The efficacy of this compensation often depends on species-specific characteristics of underlying molecular and cellular mechanisms [79].

### 3.1. Paralog-Mediated Compensation

The *DNM1* gene encodes dynamin-1, a predominantly neuronal large GTPase essential for presynaptic endocytosis and synaptic vesicle recycling during high-frequency neuronal activity [80,81]. In humans, heterozygous de novo variants in *DNM1* cause severe developmental and epileptic encephalopathy 31 (DEE31), often presenting with refractory seizures, hypotonia, and profound intellectual disability [82,83,84]. Heterozygous knock-in mice carrying the identical R237W mutation recapitulate these cellular and network abnormalities—specifically impaired endocytosis. However, they do not exhibit spontaneous tonic–clonic seizures, only stereotypical episodes of myoclonic seizures [12]. It should be emphasized that the lack of a comprehensive phenotypic assessment of dystonia, ataxia, and other motor disorders in these animals does not allow us to draw a definitive conclusion about the absence of the full spectrum of symptoms characteristic of DEE31. This incomplete phenotypic recapitulation may be attributed to functional buffering of *DNM1* within the murine nervous system. Experimental evidence indicate that compensation can be mediated by dynamin-1 paralogs: in murine neurons, *DNM3* cooperates with *DNM1* during synaptic vesicle endocytosis, while *DNM2* can sustain basal synaptic transmission and partially compensate for the loss of *DNM1* [80,85,86]. Furthermore, the impact of *DNM1* dysfunction varies across cell types, pointing to differential sensitivity among distinct neuronal populations and circuits [87]. Another factor contributing to this divergence may be differences in the temporal expression profiles of *DNM1* isoforms [88,89]. In mice, the Dnm1b isoform (containing exon 10b) is expressed predominately during early development and can partially compensate for the loss of Dnm1a, whereas the expression of Dnm1a (exon 10a) progressively increases postnatally [88,89] and is approximately twofold higher than that of Dnm1b [90]. Thus, the robust early expression of Dnm1b in mice may provide an extended developmental window for compensation. However, a direct comparative analysis of early DNM1a/DNM1b expression kinetics between humans and mice has not yet been conducted.

The *KCNQ3* gene encodes the Kv7.3 voltage-gated potassium channel subunit, which predominantly forms heteromeric channel complexes with Kv7.2. These channels generate the M-current—a key physiological mechanism that limits neuronal hyperexcitability and prevents repetitive firing [91,92]. In humans, biallelic loss-of-function variants in *KCNQ3* are associated with neonatal-onset epilepsy and severe developmental delay [93,94,95]. Conversely, constitutive or conditional loss of *Kcnq3* in mice does not induce early lethal spontaneous epilepsy or a severe systemic neurological phenotype, despite clear electrophysiological deficits, including an ~50% reduction in M-current density [96,97]. One potential explanation for this species discordance lies in differing capacities for functional compensation. Potential candidates include other Kv7 family subunits, particularly Kv7.2 and Kv7.5 (encoded by *KCNQ5*). Kv7.5 is widely expressed throughout the brain and forms functional heteromers with Kv7.3 [97,98,99]. In *Kcnq3*-deficient mouse models, residual Kv7 channel function and sensitivity to channel activators are preserved, while Kv7.2 and Kv7.5 expression levels remain largely unaltered [97]. However, direct evidence that other Kv7 subunits actively mediate this compensation remains lacking. Furthermore, potential compensatory mechanisms are not strictly rodent-specific, as *KCNQ5* is also expressed in the human cortex and hippocampus [100,101]. Therefore, an additional explanation may stem from fundamental differences in intrinsic neuronal electrophysiology. Human and mouse cortical neurons differ markedly in intrinsic excitability, action potential kinetics, and passive membrane properties [102]. The contribution of specific ionic currents to membrane properties also varies across species; for instance, h-currents exert a substantially greater influence on membrane properties in human supragranular pyramidal neurons than in murine counterparts [103]. Consequently, an equivalent level of residual M-current may carry fundamentally distinct functional consequences in humans versus mice.

### 3.2. Modifier Gene Activation

The *SCN1A* gene encodes the Nav1.1 voltage-gated sodium channel subunit, which is particularly critical for action potential generation and propagation in GABAergic interneurons [104,105]. In humans, heterozygous pathogenic variants in *SCN1A* disrupt Nav1.1 function and cause Dravet syndrome—a severe infantile-onset epileptic encephalopathy [106,107,108]. However, the phenotype of heterozygous *Scn1a* loss in mice (*Scn1a*^+^/^−^) is heavily dependent on genetic background [107,109]. For instance, on the 129S6/SvEvTac strain background, mice exhibit virtually no overt epileptic phenotype and retain normal survival, whereas on the C57BL/6J background, they develop severe spontaneous seizures and premature mortality—a phenotype much more closely mirroring human Dravet syndrome [107,110,111]. This discrepancy between strain backgrounds is driven by genetic modifiers—variants in other genes that do not independently cause disease but modulate phenotypic severity in the presence of an *Scn1a* mutation [111]. Subsequent candidate gene mapping identified *Gabra2*, encoding the α2 subunit of the GABA_A receptor, as a major modifier locus [108,111]. The C57BL/6J background harbors a specific intronic deletion in *Gabra2* that causes an approximately twofold reduction in mRNA and protein levels [111,112]. Deficiency of α2-containing GABA_A receptors attenuates postsynaptic GABAergic responses, thereby further compromising inhibitory control [113,114]. Combined with impaired GABAergic interneuron function due to Nav1.1 deficiency, this leads to a more profound disruption of inhibition and exacerbates the phenotype of *Scn1a*^+^/^−^ mice [111,115]. Restoring normal *Gabra2* expression significantly reduces seizure severity and improves survival [111]. Thus, the severity of the *Scn1a*^+^/^−^ phenotype is dictated not only by the primary mutation itself, but also by the genetic background, which can substantially aggravate or mitigate the consequences of Nav1.1 loss.

## 4. Anatomical Barrier of Striatal Somatotopy

In humans and non-human primates, the striatum possesses highly differentiated somatotopy and specialised circuits—including those dedicated to distal limb motor control and facial musculature—that are simplified or absent in mice. Consequently, mouse models frequently reproduce generalized striatal dysfunction (hyperactivity, stereotypies, impaired coordination) but fail to recapitulate the species-specific motor patterns characteristic of the human phenotype. A comparison of corticostriatal circuitry across humans, non-human primates, and mice using connectivity-based fingerprinting of resting-state fMRI data revealed that connections between the nucleus accumbens and lateral putamen are conserved across all three species, whereas 85% of human striatal voxels and 69% of macaque voxels have no counterpart in any murine corticostriatal circuit [19]. These voxels are concentrated in the caudate nucleus and anterior putamen—regions implicated in executive function, social behaviour, and language, forming loops that appear to be unique to primates [116]. It is precisely through these regions that topographically organized chorea of the distal limbs and facial musculature is expressed in humans. Corticostriatal projections from sensory and motor cortex are somatotopically organised, with topographic specificity decreasing progressively from sensory to motor and frontal territories [117]. In mice, this topography is present but functionally constrained, as the circuitry required for a digit- or facial-specific loop is absent in the rodent brain [117]. Behavioural testing of knockout animals confirms a genuine striatal deficit—hyperactivity, stereotypies, impaired rotarod performance—but fails to reproduce chorea per se, since its expression requires a fine-grained corticostriatal topography of distal limb representation that is not present in the mouse, despite the conservation of broader somatotopic organization [118].

*FOXG1* is a forkhead-family transcription factor specifically expressed in forebrain neural progenitors during early neurogenesis. Monoallelic mutations in *FOXG1* cause FOXG1 syndrome [119]. Clinically, the disorder manifests as severe intellectual disability, autistic features, continuous involuntary jerking movements, sleep disturbances, seizures, and irritability [120]. *Foxg1* haploinsufficiency in mice disrupts forebrain development, reducing the volume of the neocortex, hippocampus, and striatum. *Foxg1* mouse models display several phenotypes superficially resembling human FOXG1 syndrome, including increased locomotor activity in a novel environment and impaired learning or fear-context recognition [120]. Nevertheless, the continuous hyperkinetic–dyskinetic movement disorder characteristic of the human condition—distal chorea/athetosis, orofacial dyskinesias, and complex involuntary hyperkinesias—is reproduced only partially or in a simplified form, manifesting largely as coordination and gait deficits.

This limitation is directly related to interspecies differences in corticostriatal anatomy. Although mice retain the overall somatotopic organization, they lack the detailed representations necessary for complex movements of the distal parts of the limbs. For example, in humans, the kinematics of hand movements are ensured by connections between the motor cortex and the ventral part of the putamen—a region that consists of 85% voxels that have no structural analogue in the rodent brain [119,121] (Table 1).

## 5. Discussion

The varying number of examples in each section reflects the current availability of experimental data. For instance, evidence explicitly linking species differences to a lack of specific corticostriatal topography is currently restricted to a handful of well-studied models. Across all sections, we prioritized examples where the underlying mechanism for the interspecies difference has been experimentally validated, rather than simply listing cases where the human and mouse phenotypes diverge (the detailed methodology for literature search and the criteria for selecting studies are presented in Supplementary Material S1).

In conducting this synthesis, we identified a notable scarcity of functional data regarding paralogous protein divergence, as the available literature remains overwhelmingly transcriptomic [122,123,124]. Current research predominantly focuses on establishing orthology between human and rodent genes rather than dissecting how paralogs functionally diverge. A comprehensive characterization of species-specific paralog dynamics—incorporating detailed profiling of their expression patterns, cell-type specificity, developmental kinetics, and functional interchangeability—is urgently needed [125,126]. However, because such comparative mechanistic studies do not directly yield novel disease lines, they are frequently deprioritized in favor of generating new models. Nevertheless, systematically elucidating these biological constraints remains vital for neurobiology.

Investigating the causes of phenotypic non-recapitulation provides profound mechanistic insights into disease pathogenesis. For instance, researchers dissecting phenotypic discrepancies in *Scn1a*-deficient models uncovered the potent modifying effect of *Gabra2*, revealing that *SCN1A*-dependent pathology (such as Dravet syndrome) is heavily modulated by epistatic genetic interactions [108,111,114]. Disruption of *Gabra2* leads to a deficiency in GABA A receptor α2 subunits and a consequential impairment in postsynaptic inhibition—a principle directly applicable to human pathophysiology. An even clearer example is provided by *SCN8A* models: initial knock-in lines harboring patient-derived gain-of-function (GoF) variants failed to reproduce key aspects of the clinical phenotype. Only after identifying that developmental isoform switching occurs at an accelerated pace in mice relative to humans were researchers able to engineer a line harboring a mutation that appropriately shifted Nav1.6 activation timing, thereby achieving full recapitulation of the clinical phenotype [41,43].

Although we have classified these mechanisms above for conceptual clarity, the existing data is limited by the fact that in vivo these various mechanisms cannot be evaluated in isolation; there is no clear boundary between them. In *Dnm1* models, for example, incomplete phenotypic recapitulation can be attributed either to functional buffering by its paralogs *Dnm2* and *Dnm3* [80,85,86] or to the developmental predominance of the Dnm1b isoform, which provides an extended window for early compensation [88,89]. In *Kcnq3* models, the incomplete phenotype may reflect residual function of Kv7 channels, which is potentially supported by other subunits, as well as species-specific differences in neuronal excitability [97,101]. In *Scn8a* models, exon splicing is developmentally shifted toward embryonic stages in mice, yet phenotypic severity simultaneously depends on the precise domain location of the mutation [41]. Similarly, for *GRIN2A*, phenotypic divergence can stem from heteromeric receptor composition (whether the mutant GluN2A subunit co-assembles with wild-type GluN2A or GluN2B) [34] combined with an accelerated postnatal GluN2A/GluN2B developmental switch in rodents [36,37], which may prevent the accumulation of pathological signaling deficits over time.

Background strain differences also introduce substantial variability. Even substrains maintained and bred independently in different laboratories over extended periods undergo genetic drift [127,128,129,130], inevitably accumulating spontaneous mutations and novel variants that can suppress, exacerbate, or compensate for primary genetic lesions [127,131]. Predicting and controlling for these background-dependent modifications during model generation is exceptionally challenging. Indeed, one could argue that conclusions regarding the “non-recapitulation” of a given mutation strictly apply only to the specific genetic background and laboratory environment in which the model was evaluated.

Moreover, experimentally validated mechanisms fail to account for all instances of phenotypic divergence. Additional non-coding and regulatory factors likely play underappreciated roles, including species-specific enhancer architectures, 3′-untranslated region (3′-UTR) dynamics, and differential microRNA-mediated post-transcriptional regulation [132,133,134].

Since the phenotypes we are considering (epilepsy and motor neuron disorders) are caused by pathogenic variants that directly affect the functioning of ion channels (*SCN1A*, *SCN8A*, *KCNQ3*) and synaptic receptors (*GRIN2A*, *GRIN2D*), it is also necessary to take into account species-specific differences in the biophysical properties of neurons. On a biophysical level, the challenge of recapitulating epilepsy phenotypes in rodents is intrinsically linked to species differences in neuronal morphology, arborization complexity, and ion channel density distributions. Human cortical neurons feature larger somatic volumes and extensive dendritic arbors yet display unexpectedly low ion channel conductance (particularly Kv channels). Humans deviate from standard mammalian allometric scaling rules, exhibiting a cortical volume conductance approximately fourfold lower than predicted for a mammal of equivalent body size. Human dendritic arbors generate narrow, low-amplitude dendritic spikes rather than the prominent, complex calcium spikes characteristic of rodents, accompanied by high input resistance [135]. Furthermore, the inactivation curves of human Na^+^ and K^+^ channels are shifted toward more depolarized membrane potentials, increasing channel availability for activation. Human voltage-gated K^+^ currents display faster activation and recovery kinetics, whereas Na^+^ currents undergo slower, incomplete inactivation, supporting rapid action potential generation and precise input–output synchronization [13]. Consequently, lower channel densities make human neurons uniquely vulnerable to mutations that impair ion channel function, whereas mouse neurons recruit robust local compensatory mechanisms by engaging neighboring channel populations. Because human neurons generate action potentials rapidly due to these kinetic specializations, mouse neurons exhibit greater physiological “inertia” in response to equivalent genetic perturbations, leading to blunted disease phenotypes [136].

In addition, profound differences exist in interneuron cell-type composition and microcircuit architecture between mice and humans. In human temporal cortex versus mouse primary somatosensory cortex, marked layer-specific variations are observed in the density and spatial distribution of GABAergic axonal terminals impinging onto the soma and axon initial segments of pyramidal neurons [137,138,139]. The human neocortex also exhibits a threefold expansion in calretinin-expressing (CR+) interneurons compared to rodents, where parvalbumin-expressing (PV+) cells predominate [140,141]. CR+ interneurons serve a specialized neuromodulatory role, primarily targeting other GABAergic interneurons to mediate disinhibition across both human and rodent circuits [142,143]. However, the human hippocampus harbors unique CR+ cell types—such as multipolar cells at the stratum lacunosum–moleculare border of the CA1 region and enlarged dentate gyrus neurons—that are absent in rodents and perform uncharacterized functions. This expansion of CR+ interneurons reflects a significant evolutionary increase in interneuron connectivity density [144], suggesting that CR+ interneurons orchestrate a more specialized inhibitory network in the human brain [145].

Beyond neuronal and circuit specializations, fundamental metabolic divergences further limit phenotypic recapitulation. Although metabolic pathways in mouse and human brains are homologous at a core pathway level, significant species-specific differences in blood–brain barrier (BBB) transport activity, energy metabolism kinetics, and substrate utilization can attenuate disease manifestations in mice and alter therapeutic responses to antiseizure medications [146]. Pericytes—microvascular cells regulating trans-BBB transport and local metabolic homeostasis—exhibit divergent gene expression profiles between species. Human pericytes uniquely express the glutamate transporter *SLC1A3* (encoding GLAST1) and the potassium-chloride cotransporter *SLC12A7*, whereas mouse pericytes predominantly express *Slc1a5* (an amino acid transporter) and ATPase genes (*Atp1b2*, *Atp2a3*, *Atp13a5*). Anoctamin expression profiles are similarly inverted (Ano1 enriched in mice; *ANO3* in humans). These transporter variations highlight distinct BBB metabolic regulation strategies across species, confounding preclinical drug efficacy testing [10]. Furthermore, metabolic fluxes for glucose, acetate, β-hydroxybutyrate, and glutamine in human cortical slices proceed at significantly slower rates than in rodent brain slices. Conversely, human neurons demonstrate a superior capacity to convert exogenous glutamate into glutamine for GABA synthesis, whereas mouse neurons predominantly shunt glutamate into aspartate pathways. Owing to lower basal metabolic rates, human brain mitochondria consume less oxygen when utilizing succinate and exhibit higher coupled respiratory activity with pyruvate and malate compared to mouse mitochondria—critical factors to consider when using rodent in vitro preparations to model human brain pathology [147].

In recent years, an increasing body of evidence has shown that the gut microbiota can influence the progression of various disorders [148], including the manifestation of seizure activity [149]. Notably, direct comparisons of human and mouse microbiomes reveal substantial differences in gut bacterial species composition, sharing only about 10% similarity [150]. Distinct bacterial species exhibit unique metabolic profiles and differentially contribute to neuroinflammatory processes and GABA/glutamate balance [151,152]. Furthermore, this relationship is bidirectional: a primary genetic mutation can alter the composition of the microbiota, which in turn further modifies the neurological phenotype [153]. These translational differences are particularly pronounced between humans and laboratory mice, as the murine microbiota develops under standardized, environmentally restricted housing conditions [154,155]. Consequently, generating humanized microbiota models may represent a highly relevant strategy to improve the translational value of mouse models. For example, it has been demonstrated that transplanting modified microbiota from children on a ketogenic diet into mice is feasible and confers increased resistance to seizures [156].

Based on this comprehensive analysis, we outline a four-step practical framework for designing and interpreting transgenic mouse models:

Planning and in silico pre-analysis: systematically evaluate spatiotemporal expression profiles of target orthologs and paralogous family members. Identify potential compensatory pathways, select appropriate background strains while considering known genetic modifier loci, and define anatomical and circuit-level translational boundaries. Furthermore, differences between the microbiota of mice and humans, as well as the influence of housing conditions and the supplier’s diet, should be considered as potential external factors affecting the phenotype.

Construct validation: rigorously sequence all genetic constructs prior to transgenesis and validate their efficiency and targeting specificity in relevant cell line models.

In vitro and human-based alternative systems: prior to generating in vivo transgenic lines, conduct preliminary functional characterizations using human induced pluripotent stem cell (iPSC)-derived neurons, cerebral organoids, or 2D/3D human neural cultures. This enables assessment of pathogenic variants within both human and rodent genetic contexts.

The goal of this stage is to identify the primary disruption. In the brain of a living organism suffering from encephalopathy, the effects of the mutation are combined with secondary damage (caused, for example, by the seizures themselves). However, in an isolated cell culture, it is possible to precisely determine what exactly the pathogenic variant is doing at the initial stage, before these changes are mixed with the “noise” and before reorganization and compensation occur. Furthermore, such control helps to avoid misinterpretation of the results: if a cell model demonstrates the same disorder as in humans, the absence of a phenotype in rodents may indicate the activation of specific compensatory mechanisms. At the same time, it is necessary to take into account the limitations of in vitro systems: incomplete functional maturity and the absence of a vascular network.

Rigorous evaluation of negative results: non-recapitulation or absence of an expected phenotype should not be dismissed as an experimental failure; rather, it represents a critical biological finding that can expose novel mechanisms of species-specific resilience, buffering, and adaptation.

## 6. Conclusions

In summary, phenotypic non-recapitulation in mouse models of monogenic diseases arises from several distinct mechanistic drivers: spatiotemporal discordance in the expression of orthologs and their developmental isoforms, varying capacities of the nervous system to functionally compensate for genetic loss, and species-specific structural and neuroanatomical divergence—factors that present the greatest challenges to replicate, model, and control for. Rigorously dissecting these underlying mechanisms is essential not only for establishing the translational validity of rodent models but also for leveraging these insights toward the development of targeted human therapeutics. A mouse is neither a human nor a non-human primate; inherent translational limitations will always persist, including divergences in orthologous gene expression dynamics, cell-type composition ratios, motor circuit architecture, and intrinsic membrane excitability.

## Figures and Tables

**Table 1:** Differences in phenotypic recapitulation between humans and mouse models.

Gene	Function	Phenotype in Humans	Phenotype in Mice	Reasons for the Differences
*GRIN2D*	Encodes GluN2D, an (N-methyl-D-aspartate) NMDA receptor subunit with slow deactivation [20,21].	Developmental and Epileptic Encephalopathy (DEE) with refractory seizures, axial hypotonia and motor automatisms [22].	Heterozygous knock-in mice have lethal seizures. There are no motor automatisms. Ketamine causes seizures (in humans, it suppresses them) [22,23].	In adult mice, the amount of GluN2D in the striatum is significantly reduced [23,24,25]. It should be noted that pharmacological discrepancy may partly reflect experimental context [26].
*GRIN2A*	Encodes GluN2A, an NMDA receptor subunit with rapid deactivation [20,27,28].	DEE with myoclonic seizures and tonic–clonic seizures. Heterozygous variants are associated with severe spontaneous epilepsy [29].	Spontaneous seizures occur only in homozygous animals. In heterozygous mice, only hyperactivity, stereotypical behavior, and mild motor impairments are observed [29,30].	Differences may reflect cell-type-specific circuit architecture [31], genetic background [29,32], as well as age and sex [33]. And developmental receptor switching: the GluN2B-to-GluN2A transition is rapid in mice but takes years in humans [36,37].
*SCN8A*	Encodes Nav1.6, the principal sodium channel at the axon initial segment and nodes of Ranvier [38].	DEE with juvenile pharmacoresistant epilepsy and motor disturbances including dystonia, ataxia, and choreoathetosis [39,40].	Early Gain-of-Function (GoF) variants recapitulated seizures and premature lethality but failed to recapitulate motor abnormalities [38,39].	Splicing transitions from 5N to 5A and 18N to 18A occur over a shorter period in mice [42,43]. The 18N-to-18A switch is not established as the primary cause of motor discrepancies. Mutation-specific biophysical effects (N1768D/R1872W vs. T767I), genetic background, and behavioral testing may also contribute.
*SNAP25*	Encodes a core component of the SNARE complex that mediates Ca^2+^-dependent synaptic vesicle fusion with the presynaptic membrane and subsequent neurotransmitter release [44,45].	A patient carrying the rare *SNAP25* L50S missense variant presented with infantile spasms, tonic seizures beginning at 7 months of age, and developmental delay [46,47].	Heterozygous mice with the L50S amino acid substitution exhibit recurrent focal and generalized tonic–clonic seizures, severe motor and behavioral disorders, as well as high early postnatal mortality [48].	The more prolonged maturation of the human brain may provide a broader window for homeostatic adjustment of synaptic strength and intrinsic neuronal excitability [49,50,51]. The rarity of L50S and additional synonymous substitutions in the mouse model limit mechanistic conclusions [46,48].
*UBE3A*	It encodes the ubiquitin protein ligase E3A, which mediates the targeted degradation of proteins [52].	Angelman syndrome (AS): severe intellectual disability, absence of speech, epilepsy, ataxia, and hyperactivity [54].	Recapitulates microcephaly, ataxia, and seizure susceptibility, but does not faithfully reproduce the region- and cell-type-specific mosaic phenotype [56].	Although mouse models with a maternal *Ube3a* mutation show key features of Angelman syndrome, differences in how the paternal gene is turned off limit their accuracy. In mice, the paternal allele is silenced very quickly, though it stays partially active in a specific brain region called the suprachiasmatic nucleus (SCN) [56]. In contrast, this silencing happens much later in humans. Both copies of the gene remain active during early brain development, and the paternal copy only gradually turns off between weeks 10 and 17 [57,58].
*DEPDC5*	Encodes a component of the GATOR1 complex, which functions as a negative regulator of mTORC1 [59].	Heterozygous variants cause focal epilepsy and cortical malformations (for example, focal cortical dysplasia, hemimegalencephaly) with pronounced incomplete penetrance. The pathogenesis is based on the “two-hit” mechanism: hereditary predisposition (first hit) is followed by a somatic mutation (second hit), which leads to the formation of a focal epileptogenic clone with biallelic loss of function [60,61,62,63,68].	Heterozygous mice typically do not develop spontaneous seizures or overt cortical dysplasia [64,65,66,67].	The standard heterozygous mouse model reproduces only the first “hit” [61,62,63]. A second somatic mutation affecting the remaining functional allele may be required to generate a focal clone with biallelic loss of function [61,62,63,68]. Human cortical neurogenesis lasts approximately 4 months, versus 7–11 days in mice, and involves larger progenitor pools, including outer radial glia, which are sparse in mice [69,70,71,72]. Incomplete penetrance and variable expressivity further complicate comparisons.
*DNM1*	Encodes dynamin-1, a predominantly neuronal large GTPase essential for presynaptic endocytosis and synaptic vesicle recycling during high-frequency neuronal activity [80,81].	Heterozygous de novo variants cause severe developmental and epileptic encephalopathy 31 (DEE31), often presenting with refractory seizures, hypotonia, and profound intellectual disability [82,83,84].	Heterozygous knock-in mice carrying the R237W mutation recapitulate impaired endocytosis and cellular and network abnormalities but do not exhibit spontaneous tonic–clonic seizures; instead, they display stereotyped episodes of myoclonic seizures [12]. The lack of a comprehensive assessment of dystonia, ataxia, and other motor disorders does not allow for a definitive conclusion about the absence of a complete human phenotype.	Incomplete phenotypic recapitulation may be associated with compensation by *DNM3* and *DNM2* [80,85,86], as well as with differences in neuronal sensitivity and vulnerability [87]. Early expression of the Dnm1b isoform may also compensate for the loss of Dnm1a in mice, although comparative studies of expression in humans and mice have not been conducted [88,89,90].
*KCNQ3*	Encodes Kv7.3, which predominantly forms heteromeric channels with Kv7.2. These channels generate the M-current, limiting neuronal hyperexcitability and repetitive firing [91,92].	Biallelic loss-of-function variants in *KCNQ3* are associated with neonatal-onset epilepsy and severe developmental delay [93,94,95].	Constitutive or conditional *Kcnq3* loss does not cause early lethal spontaneous epilepsy or severe systemic neurological abnormalities, despite an approximately 50% reduction in M-current density [97,98].	Kv7.2 and Kv7.5 may preserve residual channel function, although direct evidence of compensation is lacking [97,98,99]. Species-specific differences in the excitability of neurons may also play a role: h-currents have a greater effect on human supragranular pyramidal neurons than on mouse counterarts [102,103].
*SCN1A*	Encodes Nav1.1, which is critical for action potential generation and propagation in GABAergic interneurons [104,105].	Heterozygous pathogenic variants in *SCN1A* disrupt Nav1.1 function and cause Dravet syndrome—a severe infantile-onset epileptic encephalopathy [106,107,108].	Heterozygous *Scn1a*^+^/^−^ mice show strain-dependent phenotypes: 129S6/SvEvTac mice have little overt epilepsy and normal survival, whereas C57BL/6J mice develop spontaneous seizures and premature mortality [107,109,111].	The strain-dependent phenotype is partially determined by the *Gabra2* modifier [108,111]. Intronic deletion in C57BL/6J mice reduces *Gabra2* expression by approximately half [111,112], weakening inhibition and exacerbating Nav1.1 deficiency [113,114,115]. Restoring *Gabra2* expression reduces the severity of seizures and increases survival [111].
*FOXG1*	Encodes a forkhead-family transcription factor expressed in forebrain neural progenitors during early neurogenesis [119].	Monoallelic mutations cause *FOXG1* syndrome, characterized by severe intellectual disability, autistic features, involuntary movements, sleep disturbances, seizures, and irritability [119,120].	The continuous hyperkinetic–dyskinetic movement disorder characteristic of the human condition is reproduced only partially or in a simplified form [120].	Differences in corticostriatal anatomy may contribute. Mice retain broad somatotopic organization but lack detailed representations for complex distal-limb movements. Human hand movements rely on motor cortex–ventral putamen connections; approximately 85% of voxels in this putaminal region have no structural analogue in rodent [119,121].

## Data Availability

No new data were created or analyzed in this study. Data sharing is not applicable to this article.

## Supplementary Materials

The following supporting information can be downloaded at: https://www.mdpi.com/article/10.3390/ijms27219916/s1.
