# Supplementary material for: One Disorder, Two Brains: Challenges in Modeling Epilepsy and Associated Movement Disorders in Mice
Source: Int J Mol Sci. 2026 Nov 5;27(21):9916. doi: 10.3390/ijms27219916 (PMC13763839; doi:10.3390/ijms27219916)
Supplement: Supplementary file 1 [file ijms-27-09916-s001.zip › ijms-4631514-supplementary.pdf]

Supplementary Material S1

S1. Study-Selection Approach

A search for relevant literature was conducted using the PubMed database. The search strategy was developed iteratively: initially, a broad search was performed, and the search queries were gradually refined.

At the first stage, two complementary strategies were used. The first focused on finding articles on epilepsy, DEEs, movement disorders, and animal models. The second strategy involved selecting models with an emphasis on translational relevance, phenotypic differences, and model limitations. The search yielded 1,395 and 469 records, respectively. After removing duplicates, 1,832 unique records remained.

At the next stage, to determine the specificity of the review, terms related to genes, genetic diseases, mutations, and pathogenic variants were added to both strategies. As a result, 740 and 177 records were obtained, respectively, and after deduplication, 898 unique records remained.

The selection of relevant studies from this pool was conducted according to the following criteria:

1. Presence of the gene or protein name in the article title;
2. Focus on the discussion of the experimental model;
3. Presence of a complex phenotype comprising a combination of epilepsy and movement disorders.

As a result of the screening, several dozen key articles were selected. The resulting sample covered most of the targeted genes. The exceptions were the *SNAP25* and *GRIN2A* genes: both were added during the writing of the review and further examination of the literature.

A targeted search was conducted for each of the finally selected genes. We analyzed gene specifics, available models, fundamental differences between humans and mice, and mechanisms contributing to the non-reproducibility of the phenotype. During the analysis, emphasis was placed on poorly studied genetic factors limiting translation: heterochrony and heterotopia during brain development, species-specific compensatory mechanisms, as well as the influence of genetic modifiers. The striatum was specifically noted as a node connecting the motor network and epileptic activity, and as a probable source of limitations when modeling complex syndromes.

Google Scholar was used as an additional source to identify publications on specific genes.

The search strategy is shown in Figure S1.

S1.1. PubMed Search A: Epilepsy, Movement Disorders, and Animal Models

The following query was used to retrieve publications combining epilepsy-related terms, movement-disorder terms, and animal-model terms:

```
(
  "Epilepsy"[Mesh]
  OR epilep*[Title/Abstract]
  OR "developmental and epileptic encephalopathy"[Title/Abstract]
  OR "epileptic encephalopathy"[Title/Abstract]
)
AND
(
  "Movement Disorders"[Mesh]
  OR "movement disorder"[Title/Abstract]
  OR "movement disorders"[Title/Abstract]
  OR dystoni*[Title/Abstract]
  OR dyskinesi*[Title/Abstract]
  OR ataxi*[Title/Abstract]
  OR myoclon*[Title/Abstract]
  OR tremor*[Title/Abstract]
  OR chorea[Title/Abstract]
)
AND
(
  "Disease Models, Animal"[Mesh]
  OR "Mice"[Mesh]
  OR mouse[Title/Abstract]
  OR mice[Title/Abstract]
  OR murine[Title/Abstract]
  OR transgenic[Title/Abstract]
  OR knockout[Title/Abstract]
  OR "knock-in"[Title/Abstract]
)
```

S1.2. PubMed Search B: Animal Models and Translational Limitations

This complementary query emphasized animal models of epilepsy and terms related to translational relevance and phenotypic differences:

```
(
  "Epilepsy"[Mesh]
  OR epilep*[Title/Abstract]
  OR "developmental and epileptic encephalopathy"[Title/Abstract]
  OR "epileptic encephalopathy"[Title/Abstract]
)
AND
(
  "Disease Models, Animal"[Mesh]
  OR "Mice"[Mesh]
  OR mouse[Title/Abstract]
  OR mice[Title/Abstract]
  OR murine[Title/Abstract]
)
AND
(
  translational[Title/Abstract]
  OR "species differences"[Title/Abstract]
  OR "clinical relevance"[Title/Abstract]
  OR "phenotypic differences"[Title/Abstract]
  OR "model limitations"[Title/Abstract]
)
```

S1.3. Genetic Refinement of Searches A and B

To increase specificity for genetic conditions, the following block was appended to Search A and Search B separately.

```
AND
(
  genetic*[Title/Abstract]
  OR monogenic[Title/Abstract]
  OR inherited[Title/Abstract]
  OR mutation*[Title/Abstract]
  OR variant*[Title/Abstract]
  OR pathogenic[Title/Abstract]
)
```

S1.4. Gene-Specific PubMed Search D

For targeted assessment of individual genes, GENE_SYMBOL was replaced with the relevant gene symbol and the query was run separately for each target gene. The search combined species/model terms with terms concerning cross-species differences, development, gene expression, compensation, modifiers, and striatal mechanisms:

```
(
  GENE_SYMBOL[Title/Abstract]
)
AND
(
  mouse[Title/Abstract]
  OR mice[Title/Abstract]
  OR murine[Title/Abstract]
  OR human[Title/Abstract]
  OR humans[Title/Abstract]
)
AND
(
  "species differences"[Title/Abstract]
  OR "cross-species"[Title/Abstract]
  OR ortholog*[Title/Abstract]
  OR "gene expression"[Title/Abstract]
  OR developmental[Title/Abstract]
  OR spatiotemporal[Title/Abstract]
  OR "alternative splicing"[Title/Abstract]
  OR isoform*[Title/Abstract]
  OR compensat*[Title/Abstract]
  OR paralog*[Title/Abstract]
  OR "genetic modifier"[Title/Abstract]
  OR "genetic modifiers"[Title/Abstract]
  OR "translational relevance"[Title/Abstract]
  OR "phenotypic differences"[Title/Abstract]
  OR "functional redundancy"[Title/Abstract]
  OR striat*[Title/Abstract]
  OR corticostriatal[Title/Abstract]
)
```

S1.5. Supplementary Google Scholar Search

Google Scholar was used as a supplementary source for targeted gene-specific searches. The following query template was adapted for each gene by replacing GENE_SYMBOL:

```
GENE_SYMBOL mouse human ("species differences" OR developmental OR expression OR splicing OR compensation
OR "genetic modifier")
```

The individual yields from the gene-specific PubMed and Google Scholar searches were not separately tabulated; these searches supplemented the broad strategies described above.

S2. Flow Diagram For Literature Search

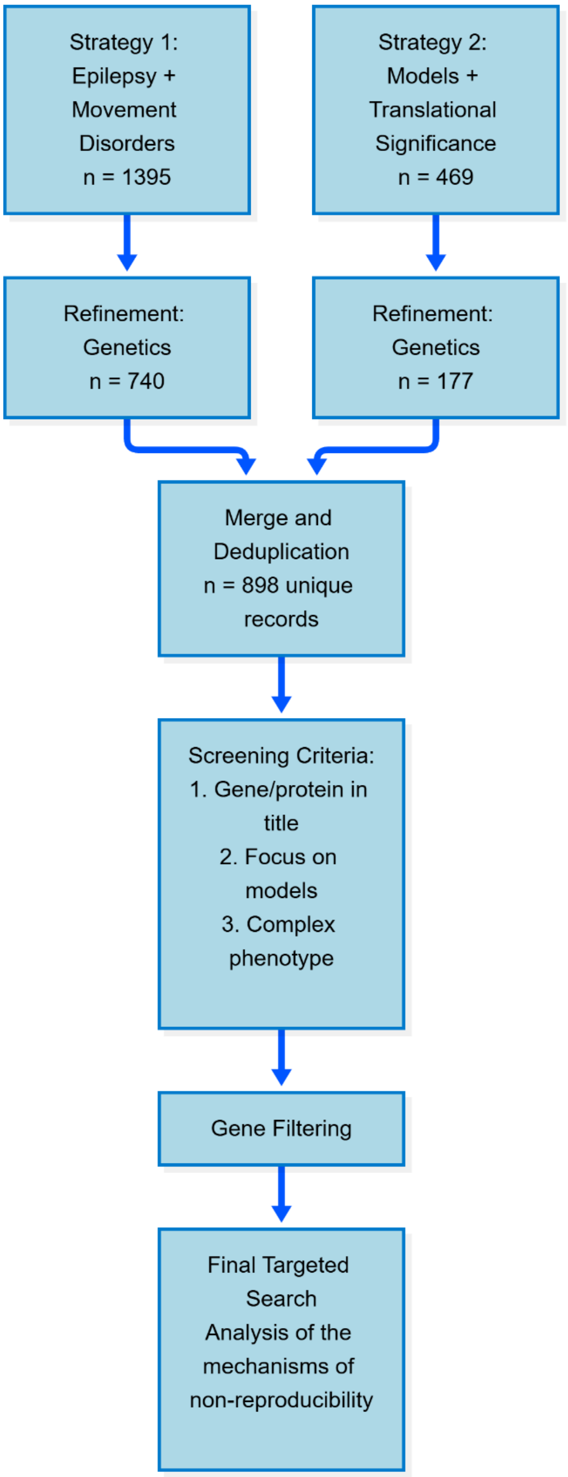

Figure S1. Flow diagram of literature selection.

S2.1 Mermaid Code For The Flow Diagram.

```
graph TD
    classDef default fill:lightblue,stroke:#007acc,stroke-width:2px,color:black;
    linkStyle default stroke:#0055ff,stroke-width:4px;

    A[Strategy 1: Epilepsy + Movement Disorders<br>n = 1395] --> C[Refinement: Genetics<br>n = 740]
    B[Strategy 2: Models + Translational Significance<br>n = 469] --> D[Refinement: Genetics<br>n = 177]
    C --> E[Merge and Deduplication<br>n = 898 unique records]
    D --> E
    E --> F[Screening Criteria:<br>1. Gene/protein in title<br>2. Focus on models<br>3. Complex phenotype]
    F --> G[Gene Filtering]
    G --> H[Final Targeted Search<br>Analysis of the mechanisms of non-reproducibility]
```
